# How to Boost the Activity of the Monolayer Pt Supported on TiC Catalysts for Oxygen Reduction Reaction: A Density Functional Theory Study

**DOI:** 10.3390/ma12091560

**Published:** 2019-05-13

**Authors:** Hui Zhu, Houyi Liu, Lei Yang, Beibei Xiao

**Affiliations:** School of Energy and Power Engineering, Jiangsu University of Science and Technology, Zhenjiang 212003, China; zhjust16@163.com (H.Z.); hyliu17@163.com (H.L.); yljust18@163.com (L.Y.)

**Keywords:** oxygen reduction reaction, DFT calculation, core-shell structure

## Abstract

Developing the optimized electrocatalysts with high Pt utilization as well as the outstanding performance for the oxygen reduction reaction (ORR) has raised great attention. Herein, the effects of the interlayer ZrC, HfC, or TiN and the multilayer Pt shell on the adsorption ability and the catalytic activity of the TiC@Pt core-shell structures are systemically investigated by density functional theory (DFT) calculations. For the sandwich structures, the presence of TiN significantly enhances the adsorption ability of the Pt shell, leading to the deterioration of the activity whilst the negligible influence of the ZrC and HfC insertion results the comparable performance with respect to TiC@Pt_1ML_. In addition, increasing the thickness of the Pt shell reduces the oxyphilic capacity and then mitigates the OH poisoning. From the free energy plots, the superior activity of TiC@Pt_2ML_ is identified in comparison with 1ML and 3ML Pt shell. Herein, the improved activity with its high Pt atomic utilization makes the potential TiC@Pt_2ML_ electrocatalyst for the future fuel cells.

## 1. Introduction

Proton exchange membrane fuel cells (PEMFC) have attracted widespread attention due to their high efficient and zero carbon emission for the hydrogen economy [1,2,3,4]. To accelerate the sluggish kinetics of the oxygen reduction reaction (ORR) at the cathode, commercial catalysts consist of platinum deposited on a carbon support [5]. However, the high loading of the Pt catalysts results in a major challenge for future commercialization [6]. In this regard, the development of efficient catalysts with reduced Pt content is of great importance. 

The core-shell structure with a non-Pt core can significantly improve the Pt utilization, thereby reducing the Pt content and, thus, the cost [7,8]. It as previously revealed that when transition metal (TM) elements acted as the core, such as Pd [9,10], Ru [11], or Ir [12], the ORR activity of the corresponding TM@Pt core-shell was enhanced compared with the commercial Pt/C. However, such a TM core would not be suitable from an economic aspect [13]. On the other hand, titanium carbide (TiC) is a good alternative of Pt due to its similar electronic structure [14], being important in the field of catalysis. As reported, the TiC supported Pt catalysts possess the enhanced performance of the methanol oxidation reaction, hydrogen evolution reaction, and ORR, implying the positive effect of TiC [15,16,17,18]. It is believed that the TiC core could modify the electronic structure of the corresponding Pt shell to boost the ORR performance. Therefore, the core-shell structure consisting of the TiC core and the Pt shell acting as the ORR cathode could be the solution for the future requirements of the PEMFC cathode material. 

TiC suffers from stability degradation due to oxide formation during the electrochemical cycles [19]. To settle the issue, increasing the thickness of the Pt shell is a viable strategy to protect the core [20,21,22]. The durability enhancement of the Pd@Pt catalysts with multilayer Pt shells provides the direct evidence [9]. In addition, the sandwich structure created by inserting an interlayer would be another good solution [23], which is easily achieved by the controllable synthesis benefit from the experimental development. As reported, the robust stability of the ZrC and HfC are of great potential to resist the electrochemical corrosion, besides the TiC support, being favorable as support materials in the harsh conditions [24]. Furthermore, the efficient and durable TiN materials are also merged due to the passivation degree by oxygen [25,26]. Therefore, ZrC, HfC, as well as TiN, would be suitable selections to act as the interlayer. Since the different electronic effects caused by the shell thickness and the interlayer would modulate the ORR activity of TiC@Pt [19,27,28,29], the systematic influences of the aforementioned factors on the ORR activity of TiC@Pt core-shell material are as yet untouched, raising our interest.

In the manuscript, density functional theory (DFT) calculations are used within an electrochemical framework to analyze the ORR electrocatalysis on the TiC@Pt core-shell materials and their derivatives. The adsorption behavior of the intermediates is calculated, for the evaluation of the scaling relationship and then thermodynamically free energy. The data provides the fundamental understanding of relationship between the activity of TiC@Pt core-shell materials and the interlayer or the shell thickness and further identify the optimal candidate to guide the experimental progress for top-down material design.

## 2. The Calculation Details

All calculations are performed within the DFT framework as implemented in DMol^3^ code [30,31]. The generalized gradient approximation with the Perdew–Burke–Ernzerhof functional (GGA–PBE) is employed to describe exchange and correlation effects [32]. The DFT semi-core pseudopots (DSPP) core treatment is implemented for relativistic effects, which replace core electrons by a single effective potential and introduce some degree of relativistic correction into the core [33]. The double numerical atomic orbital augmented by a polarization function is chosen as the basis set [30]. Herein, the PBE/DNP combination in Dmol^3^ code has been widely employed for the ORR electrocatalysis [7,8,34,35,36]. Furthermore, these parameters have been used for the TiC@Pt or TiN@Pt system [37]. Our calculation method is consistent with the previous works, indicating the feasibility. A smearing of 0.005 Ha (1 Ha = 27.21 eV) to the orbital occupation is applied to achieve accurate electronic convergence. The spin-unrestricted method is used for all calculations. The minimum energy paths for the ORR were obtained by the LST/QST tools in the DMol^3^ code. 

**Figure 1:**
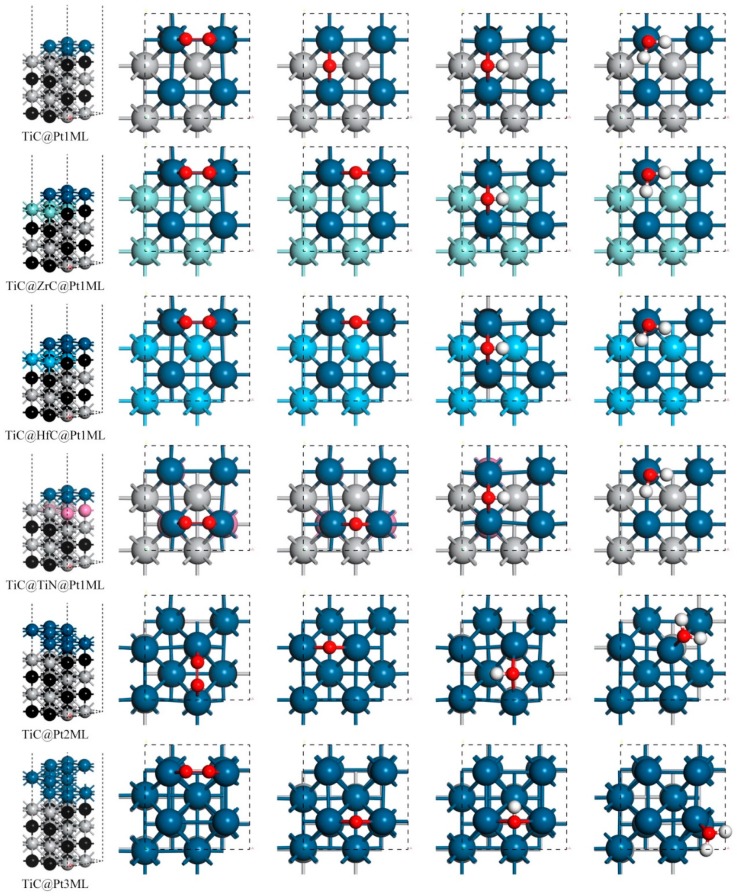
The catalyst structures and the stable adsorption configurations.

The TiC@Pt(001) surfaces are modeled as periodically repeated 2 × 2 supercell. A 15 Å-thick vacuum is added along the direction perpendicular to the surface to avoid the artificial interactions between slab and its images. The corresponding structure of TiC@Pt(001) and its derivatives are schematically illustrated in Figure 1. In all of the structure optimization calculations, the atoms in the bottom two layers are fixed while other are fully relaxed.

The adsorption energies *E*_ads_(M) are calculated by the following equations:

*E*_ads_(M) = *E*_M/slab_ − (*E*_M_ + *E*_slab_) (1)

where *E*_M/slab_, *E*_M_, and *E*_slab_ are the energies of the adsorption systems, the ORR intermediates and the catalyst, respectively. 

Gibbs free energy changes (∆*G*) of the ORR elemental steps have been calculated according to the computational hydrogen electrode (CHE) model developed by Nørskov et al. where the chemical potential of proton/electron (H+ + e^−^) in solution is equal to the half of the chemical potential of a gas-phase H_2_ [5]. The ∆G for every elemental step can be determined as following:

∆*G* = ∆*E* + ∆ZPE − T∆*S* + ∆*G*pH + ∆*GU* (2)

where ∆*E* is the electronic energy difference based on DFT calculations, ∆ZPE is the change in zero point energy, T is the temperature (equal to 298.15 K here), ∆*S* is the change in the entropy, and ∆*G*pH and ∆*GU* are the free energy contributions due to variation in pH value (pH is set as 0 in acid medium) and electrode potential U, respectively. In order to decrease the calculation consumption, the approximate correction ∆ZPE − T∆*S* to ∆*E* (0.05/0.35 eV of O*/OH*) are used for constructed the ∆*G* [5].

## 3. Results and Discussion

**Figure 2:**
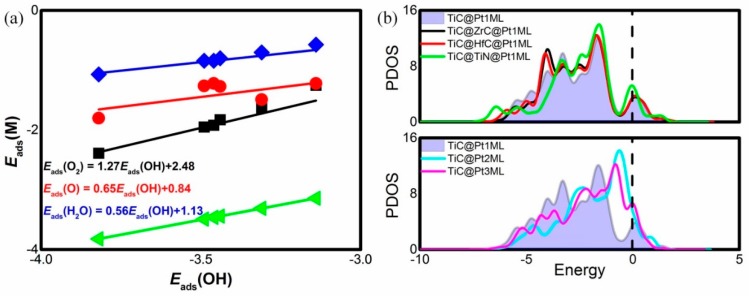
(**a**) The adsorption energy *E*_ads_ of the ORR intermediates as a function of *E*_ads_ (OH); and (**b**) the partial density of states (PDOS) for the Pt surface atoms.

In order to characterize the adsorption ability, the high-symmetry adsorption sites are considered, including the top, bridge, and hollow sites [7,38]. The favorable adsorption sites are shown in Figure 1 and the corresponding adsorption energies *E*_ads_ are listed in Table 1. For TiC@Pt_1ML_, the favorable adsorption site of O_2_ is the bridge site with the *E*_ads_(O_2_) of −1.83 eV, indicating the efficiency of the O–O activation, in line with the previous work [39]. Similarly, the O and OH are located at the bridge sites with the *E*_ads_ of −1.27 and −3.44 eV, respectively. The product H_2_O is suited at the top site and the *E*_ads_(H_2_O) is −0.80 eV, being stronger than the solvation stabilization energy of bulk H_2_O (about −0.40 eV) [40]. Comparing the data of *E*_ads_(O_2_) and *E*_ads_(H_2_O), the product H_2_O is readily replaced by the reactant O_2_ for the next ORR cycle. For the TiC@ZrC@Pt_1ML_ or TiC@HfC@Pt_1ML_, the difference of the *E*_ads_ is less than −0.05 eV with respect to the TiC@Pt_1ML_, indicating the negligible effects of the ZrC or HfC interlayer on the adsorption behavior. However, the binding between the Pt shell and the adsorbates is significantly enhanced by inserting TiN interlayer. Reserving the stable adsorption sites, the corresponding *E*_ads_ are −2.39, −1.80, −3.82, and −1.07 eV for O_2_, O, OH and H_2_O, respectively. Herein, the presence of TiN boost the oxyphilic ability compared with TiC@Pt_1ML_. Such adsorption variation is reasonable that the interaction between the Pt shell and the substrate of the TiC@TiN@Pt_1ML_ is via the Pt–N bonds, being different from the Pt–C bonds for TiC@Pt_1ML_. On the other hand, increasing the Pt shell thickness would weaken the ligand effect of the TiC core, changing the adsorption capability [11]. As listed in Table 1, the *E*_ads_ of O_2_, OH and H_2_O are decreased to −1.25, −3.14, and −0.57 eV, while *E*_ads_(O) is slightly disturbed with the value of −1.22 eV for the TiC@Pt_2ML_ whilst the corresponding *E*_ads_ of the TiC@Pt_3ML_ are −1.63, −1.49, −3.31, and −0.70 eV for the O_2_, O, OH, and H_2_O adsorption, respectively. That is, the multilayer Pt shell weakens the O_2_, OH and H_2_O adsorption besides O affinity referred to 1ML Pt system, with the *E*_ads_ order of TiC@Pt_1ML_ > TiC@Pt_3ML_ > TiC@Pt_2ML_. Due to the *E*_ads_ dependence, it is implied that the ORR activity could be tuned by the interlayer and the Pt thickness. Herein, the *E*_ads_ of the ORR intermediates as a function of *E*_ads_(OH) is established in Figure 2a. As shown, the scaling relationship is clearly observed, in consistence with the previous results [41]. That is:

*E*_ads_(O_2_) = 1.27*E*_ads_(OH) + 2.48 (3)

*E*_ads_(O) = 0.65*E*_ads_(OH) + 0.84 (4)

*E*_ads_(H_2_O) = 0.56*E*_ads_(OH) + 1.13 (5)

As is well-known, the adsorption strength is correlated with the *d* band center of the catalysts according to the *d* band model where the higher (lower) of the *d* band center referred to the Fermi energy generally corresponds to the stronger (weaker) adsorption ability [42]. Herein, in order to understand the physical origin of the *E*_ads_ change, the *d* partial density of states (PDOS) of the Pt surface is plotted in Figure 2b. As shown in the top panel, the *d* orbital of the sandwich structures are altered by the different interlayers. Therein, the *d* bands are almost overlapped for the TiC@ZrC@Pt_1ML_ and TiC@HfC@Pt_1ML_ while the obvious upshift is observed for TiC@TiN@Pt_1ML_. Quantitatively, the *d* band centers are calculated and listed in Table 2. The corresponding values are −2.78, −2.80, and −2.40 eV for the mentioned systems, respectively. That is, the enhanced adsorption ability of TiC@TiN@Pt_1ML_ is attributed by the robustness of the *d* electrons. Conversely, the *d* band model is unfeasible for the multilayer Pt shell. In the bottom panel of Figure 2b, the *d* orbital of TiC@Pt_2ML_ and TiC@Pt_3ML_ are obviously moved toward the Fermi energy with respect to TiC@Pt_1ML_. As the Pt thickness increases from 1ML to 2ML and 3ML, the corresponding *d* band centers are changed from −2.85 to −1.83 and −2.05 eV, respectively. That is, the *d* band center follows the order of TiC@Pt_2ML_ > TiC@Pt_3ML_ > TiC@Pt_1ML_, being contrary against the *E*_ads_(OH) tendency. Herein, the higher *d* band centers is accompanied by the weaker *E*_ads_(OH), being deviated from the *d* band model [43]. In order to explain the abnormal phenomenon, the Mulliken charge is analyzed. As shown in Table 2, the charge transferred from the TiC core to Pt shell is reduced as the thickness increases, indicating Pt shell trends to be electronic neutrality. It implies that the electrostatic repulsion between the multilayer Pt shell and the OH would be lessened. Herein, the charge transformation is unaccountable for the *E*_ads_(OH) variation. As previous revealed, the adsorption energy is divided into the interaction energy and the deformation energy where the endothermic latter leads to the energetically loss of the adsorption energy [44]. Therefore, the geometrical factors are considered where the average bond length of the Pt–Pt bonds before and after OH adsorption (*D*_bef_ and *D*_aft_) are given in Table 2. As shown, no significant change occurs during the OH attachment. However, the Pt–Pt bond underlying the adsorbed OH are elongated with the values of 3.32 and 3.28 Å for TiC@Pt_2ML_ and TiC@Pt_3ML_, compared with the shortened 2.80 Å for the TiC@Pt_1ML_, respectively. Plausibly, it is inferred that the deviation from the *d* band model is attributed from the catalysts deformation [44]. 

Due to the scaling relationship, the optimization prerequisite of the electrocatalysts is located at the trade-off adsorption ability since too strong leads to the poisoning and too weak implies the insufficient capture [45,46]. To evaluate the activity, the simple O_2_ dissociation are taken into consideration with the elemental steps listing in the following according to the previous report [38]. Due to the small kinetic barrier of proton transfer, which could be ignored at the high potential [47,48], our attentions are focused on the free energies *G* based on the computational hydrogen model [5]:

1/2O_2_ + * → O^*^ (6)

O^*^ + (H^+^ + *e*^−^) → HO^*^ (7)

HO^*^ + (H^+^ + *e*^−^) → H_2_O + * (8)

**Figure 3:**
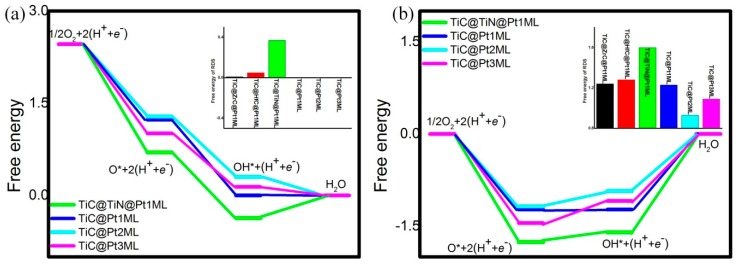
The free energies at the potential of 0 V (**a**) and 1.23 V (**b**). The RDS Δ*G* are shown in the insets.

Figure 3 describes the reaction process at the potential U of 0 V and 1.23 V, respectively. The corresponding free energies change Δ*G* are summarized in Table 3 where the positive (negative) Δ*G* means the endothermic (exothermic) reaction. For TiC@Pt_1ML_ at the potential of 0 V, the O_2_ dissociation and the OH formation are exothermic processes with the Δ*G* values of −1.23 and −1.23 eV, respectively. Meanwhile, the H_2_O formation from OH protonation is energetically balanced with the Δ*G* of 0 eV. Due to the potential-dependence, at U = 1.23 V, the Δ*G* of the OH formation and H_2_O formation are increased to 0 and 1.23 eV, respectively. Thus, the rate-determining step (RDS) of TiC@Pt_1ML_ is located at the H_2_O formation. Based on the data in Table 3, the similar situation is found for the sandwich structures. The RDS are reserved at the final step of H_2_O formation with the Δ*G* of 0.01, 0.05 and 0.37 eV at U = 0 V or 1.24, 1.28, and 1.60 eV at U = 1.23 V for inserting ZrC, HfC, and TiN interlayer, respectively. Herein, no activity improvement is achieved in comparison with TiC@Pt_1ML_. On the other hand, being different from TiC@Pt_1ML_, the elemental steps of the TiC@Pt_2ML_ at U = 0 V are energetically downward with the Δ*G* of −1.18, −0.98, and −0.30 eV for the O_2_ dissociation, the OH formation and the H_2_O formation, respectively. At U = 1.23 V, the OH formation and H_2_O formation are changed to be endothermic and the corresponding Δ*G* are 0.25 and 0.93 eV, indicating the RDS is reserved as the H_2_O formation. Analogously, for 3ML Pt shell, the RDS of H_2_O formation with the Δ*G* of −0.14 eV at U = 0 V and 1.09 eV at U = 1.23 V are observed. Herein, from the thermodynamic aspect, the promotion effect on the ORR activity is observed for the multilayer Pt shell. 

**Figure 4:**
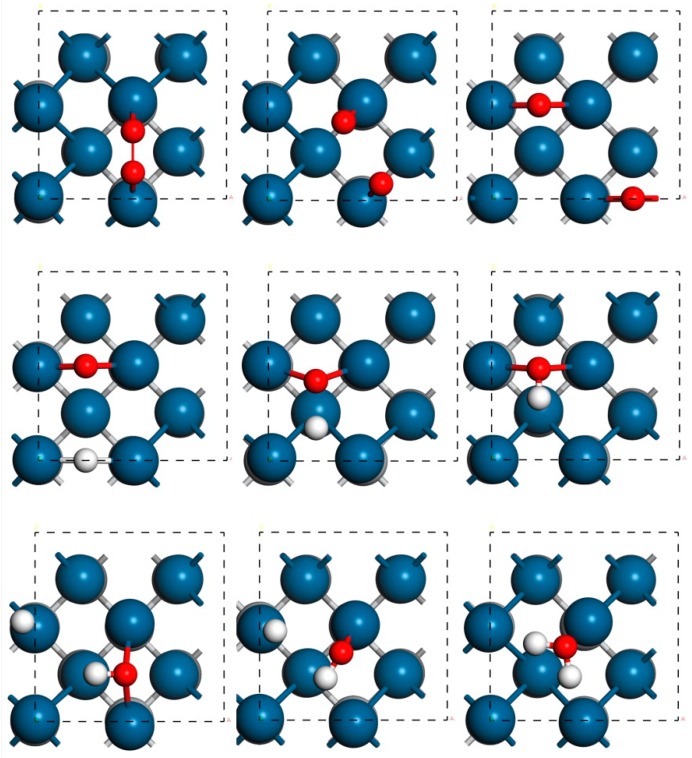
The reaction pathway of the O_2_ dissociation mechanism on TiC@Pt_2ML_.

In addition to the thermodynamic analysis, the kinetic barriers of the O_2_ dissociation mechanism on TiC@Pt_2ML_ are further considered. The reaction pathway of TiC@Pt_2ML_ is plotted in Figure 4. The corresponding reaction barriers *E*_a_ and reaction energy *E*_r_ are tabulated in Table 4. Herein, TiC@Pt_1ML_ and TiC@TiN@Pt_1ML_ are selected as references. For O_2_ splitting into the O atoms, the *E*_a_ of TiC@Pt_2ML_ is 1.28 eV, being slightly higher than 1.05 eV of TiC@Pt_1ML_ and 0.76 eV of TiC@TiN@Pt_1ML_. The weaker *E*_ads_(O_2_) correlates to the higher *E*_a_ of O_2_ dissociation, indicating the degradation of O_2_ activation, in consistence with the previous reports [39,49]. Noting that the O_2_ dissociation on TiC@Pt_1ML_ would be significantly boosted by lowing O_2_ coverage where the *E*_a_ reduces from 0.86 eV to 0.36 eV as the O_2_ coverage changes from 1/4 ML to 1/9 ML [39]. Therefore, it is reasonably believed that the mentioned phenomenon is occurred on TiC@Pt_2ML_, implying that the barrier of O_2_ splitting would be overcome at the room temperature [50]. Furthermore, the similar situation is observed for the OH formation where the unfeasibility of TiC@Pt_2ML_ is identified compared with TiC@Pt_1ML_ and TiC@TiN@Pt_1ML_. However, the *E*_a_ of the H_2_O formation is 0.91, 1.04, and 1.41 eV for TiC@Pt_2ML_, TiC@Pt_1ML_, and TiC@TiN@Pt_1ML_, respectively. The lower value implies that the OH hydrogenation is speeded by the presence of TiC@Pt_2ML_. Herein, the kinetic benefit of TiC@Pt_2ML_ is confirmed that the low oxyphilic character avails the OH poisoning, in line with the thermodynamic data [51]. 

Noting that he experimental verification should be urgently needed to confirm the DFT prediction. Herein, we believe our results realizable due to the following reasons: firstly, CHE model has been successfully applied to interpret the experimental data and design the novel electrocatalysts for metal, oxides as well as carbon-based materials [41,52,53,54,55,56,57,58]; secondly, the development of the synthesis technology leads to the feasibility of the TiC@Pt materials with different core composition as well as shell thickness [17,59,60,61]. Therefore, it is reasonably believed that the DFT candidate of TiC@Pt_2ML_ materials could be experimentally achieved. 

## 4. Conclusions

In this study, DFT calculation is used to investigate the effect of the interlayer and shell thickness on ORR activity. Compared with the TiC@Pt_1ML_, the comparable adsorption ability was found for TiC@ZrC@Pt_1ML_ and TiC@HfC@Pt_1ML_ whilst the presence of TiN causes a sharp enhancement of the adsorption energy. From the PDOS analysis, the upshifted *d* band of TiC@TiN@Pt_1ML_ supports the variation of the adsorption behavior. On the other hand, the multiplayer Pt shell generally weakens the oxyphilic affinity with the order of TiC@Pt_1ML_ > TiC@Pt_3ML_ > TiC@Pt_2ML_. The deviation from the famous *d* band model is plausibly attributed from the structural deformation. Furthermore, the RDS of the considered systems are identified as the H_2_O formation. The decrease of the adsorption capacity alleviates the OH poisoning and boosts the ORR activity. Herein, the enhanced activity of the TiC@Pt_2ML_ is confirmed compared with TiC@Pt_1ML_. The promising ORR performance of the multilayer Pt supported on TiC supplies the theoretical guide for the synthesis.

## Figures and Tables

**Table 1:** The corresponding adsorption energy *E*_ads_ of possible ORR intermediates.

Catalyst System	*E*_ads_(O_2_)	*E*_ads_(O)	*E*_ads_(OH)	*E*_ads_(H_2_O)
TiC@Pt_1ML_	−1.83	−1.27	−3.44	−0.8
TiC@ZrC@Pt_1ML_	−1.92	−1.22	−3.46	−0.84
TiC@HfC@Pt_1ML_	−1.95	−1.26	−3.49	−0.84
TiC@TiN@Pt_1ML_	−2.39	−1.80	−3.82	−1.07
TiC@Pt_2ML_	−1.25	−1.22	−3.14	−0.57
TiC@Pt_3ML_	−1.63	−1.49	−3.31	−0.70

**Table 2:** The *d* band center and the Mulliken charge of the Pt surface atom. *D*_bef_ and *D*_aft_ stand for the average bond length of the surficial Pt–Pt bonds before and after OH adsorption while *d* is the Pt–Pt bond length underlying the OH species.

Catalyst System	*d* Band Center	Mulliken Charge	*D*_bef_	*D*_aft_	*d*
TiC@Pt_1ML_	−2.85	−0.225	3.04	3.01	2.80
TiC@ZrC@Pt_1ML_	−2.78	−0.275	3.06	3.00	2.81
TiC@HfC@Pt_1ML_	−2.80	−0.275	3.06	3.00	2.80
TiC@TiN@Pt_1ML_	−2.40	−0.108	3.07	3.00	2.75
TiC@Pt_2ML_	−1.83	−0.107	3.06	3.09	3.32
TiC@Pt_3ML_	−2.05	−0.029	2.93	3.07	3.28

**Table 3:** The free energy change Δ*G* at the potential of 0 V and 1.23 V.

Catalyst System	U = 0 V			U = 1.23 V		
	R1	R2	R3	R1	R2	R3
TiC@Pt_1ML_	−1.23	−1.23	0	−1.23	0	1.23
TiC@ZrC@Pt_1ML_	−1.18	−1.29	0.01	−1.18	−0.06	1.24
TiC@HfC@Pt_1ML_	−1.22	−1.29	0.05	−1.22	−0.06	1.28
TiC@TiN@Pt_1ML_	−1.76	−1.07	0.37	−1.76	0.16	1.60
TiC@Pt_2ML_	−1.18	−0.98	−0.30	−1.18	0.25	0.93
TiC@Pt_3ML_	−1.45	−0.87	−0.14	−1.45	0.36	1.09

R1: 1/2O_2_ + * → O^*^; R2: O^*^ + (H^+^ + *e*^−^) → HO^*^; R3: HO^*^ + (H^+^ + *e*^−^) → H_2_O + *.

**Table 4:** The reaction barriers *E*_a_ and reaction energy *E*_r_ of the O_2_ dissociation mechanism.

Catalyst System	O_2_→2O		O + H→OH		OH + H→H_2_O	
	*E*_a_	*E*_r_	*E*_a_	*E*_r_	*E*_a_	*E*_r_
TiC@Pt_1ML_	1.05	−0.30	0.46	−1.08	1.04	−0.22
TiC@TiN@Pt_1ML_	0.76	−0.70	0.55	−0.90	1.41	0.68
TiC@Pt_2ML_	1.28	−0.97	0.69	−0.46	0.91	0.04

## References

[1] Stacy J., Regmi Y.N., Leonard B., Fan M. (2017). The recent progress and future of oxygen reduction reaction catalysis: A review. Renew. Sustain. Energy Rev..

[2] Chen D., Xu Y., Tade M.O., Shao Z. (2017). General Regulation of Air Flow Distribution Characteristics within Planar Solid Oxide Fuel Cell Stacks. ACS Energy Lett..

[3] Wei T., Zhang Z., Zhu Z., Zhou X., Wang Y., Wang Y., Zhuang Q. (2019). Recycling of waste plastics and scalable preparation of Si/CNF/C composite as anode material for lithium-ion batteries. Ionics.

[4] Zhang L., Cai Z., Yao Z., Ji L., Sun Z., Yan N., Zhang B., Xiao B., Du J., Zhu X.-Q. (2019). A striking catalytic effect of facile synthesized ZrMn_2_ nanoparticles on the de/rehydrogenation properties of MgH_2_. J. Mater. Chem. A.

[5] Nørskov J.K., Rossmeisl J., Logadottir A., Lindqvist L., Kitchin J.R., Bligaard T., Jónsson H. (2004). Origin of the Overpotential for Oxygen Reduction at a Fuel-Cell Cathode. J. Phys. Chem. B.

[6] Debe M.K. (2012). Electrocatalyst approaches and challenges for automotive fuel cells. Nat. Cell Boil..

[7] Xiao B.B., Jiang X.B., Jiang Q. (2016). Density functional theory study of oxygen reduction reaction on Pt/Pd_3_Al(111) alloy electrocatalyst. Phys. Chem. Chem. Phys..

[8] Xiao B.B., Jiang X.B., Yang X.L., Zheng F. (2016). The segregation resistance of the Pt_2ML_/Os/Pd_3_Al sandwich catalyst for oxygen reduction reaction: a density functional theory study. Phys. Chem. Chem. Phys..

[9] Xie S., Choi S.-I., Lu N., Roling L., Herron J.A., Zhang L., Park J., Wang J., Kim M.J., Xie Z. (2014). Atomic Layer-by-Layer Deposition of Pt on Pd Nanocubes for Catalysts with Enhanced Activity and Durability toward Oxygen Reduction. Nano Lett..

[10] Choi R., Choi S.-I., Choi C.H., Nam K.M., Woo S.I., Park J.T., Han S.W.H. (2013). Designed synthesis of well-defined Pd@Pt core-shell nanoparticles with controlled shell thickness as efficient oxygen reduction electrocatalysts. Chem. A Eur. J..

[11] Yang L., Vukmirovic M.B., Su D., Sasaki K., Herron J.A., Mavrikakis M., Liao S., Adzic R.R. (2013). Tuning the Catalytic Activity of Ru@Pt Core–Shell Nanoparticles for the Oxygen Reduction Reaction by Varying the Shell Thickness. J. Phys. Chem. C.

[12] Todoroki N., Watanabe H., Kondo T., Kaneko S., Wadayama T. (2016). Highly Enhanced Oxygen Reduction Reaction Activity and Electrochemical Stability of Pt/Ir(111) Bimetallic Surfaces. Electrochim. Acta.

[13] Tian X., Tang H., Luo J., Nan H., Shu T., Du L., Zeng J., Liao S., Adzic R.R. (2017). High-Performance Core–Shell Catalyst with Nitride Nanoparticles as a Core: Well-Defined Titanium Copper Nitride Coated with an Atomic Pt Layer for the Oxygen Reduction Reaction. ACS Catal..

[14] Hwu H.H., Chen J.G. (2005). Surface Chemistry of Transition Metal Carbides. ChemInform.

[15] Ou Y., Cui X., Zhang X., Jiang Z. (2010). Titanium carbide nanoparticles supported Pt catalysts for methanol electrooxidation in acidic media. J. Power Sources.

[16] Park H.-U., Lee E., Kwon Y.-U. (2017). TiC supported Pt-based nanoparticles: Facile sonochemical synthesis and electrocatalytic properties for methanol oxidation reaction. Int. J. Hydrogen Energy.

[17] Kimmel Y.C., Yang L., Kelly T.G., Rykov S.A., Chen J.G. (2014). Theoretical prediction and experimental verification of low loading of platinum on titanium carbide as low-cost and stable electrocatalysts. J. Catal..

[18] Chiwata M., Kakinuma K., Wakisaka M., Uchida M., Deki S., Watanabe M., Uchida H. (2015). Oxygen Reduction Reaction Activity and Durability of Pt Catalysts Supported on Titanium Carbide. Catalysts.

[19] Roca-Ayats M., García G., Galante J., Peña M.A., Martínez-Huerta M., Huerta M.V.M. (2014). Electrocatalytic stability of Ti based-supported Pt_3_Ir nanoparticles for unitized regenerative fuel cells. Int. J. Hydrogen Energy.

[20] Escudero-Escribano M., Verdaguer-Casadevall A., Malacrida P., Grønbjerg U., Knudsen B.P., Jepsen A.K., Rossmeisl J., Stephens I.E.L., Chorkendorff I. (2012). Pt_5_Gd as a Highly Active and Stable Catalyst for Oxygen Electroreduction. J. Am. Chem. Soc..

[21] Stamenkovic V.R., Mun B.S., Arenz M., Mayrhofer K.J.J., Lucas C.A., Wang G., Ross P.N., Marković N.M. (2007). Trends in electrocatalysis on extended and nanoscale Pt-bimetallic alloy surfaces. Nat. Mater..

[22] Kuttiyiel K.A., Sasaki K., Choi Y., Su D., Liu P., Adzic R.R. (2012). Nitride stabilized PtNi core-shell nanocatalyst for high oxygen reduction activity. Nano Lett..

[23] Escaño M.C.S. (2015). First-principles calculations of the dissolution and coalescence properties of Pt nanoparticle ORR catalysts: The effect of nanoparticle shape. Nano Res..

[24] Regmi Y.N., Waetzig G.R., Duffee K.D., Schmuecker S.M., Thode J.M., Leonard B.M. (2015). Carbides of group IVA, VA and VIA transition metals as alternative HER and ORR catalysts and support materials. J. Mater. Chem. A.

[25] Seifitokaldani A., Savadogo O., Perrier M. (2014). Density Functional Theory (DFT) Computation of the Oxygen Reduction Reaction (ORR) on Titanium Nitride (TiN) Surface. Electrochim. Acta.

[26] Kim H., Cho M.K., Kwon J.A., Jeong Y.H., Lee K.J., Kim N.Y., Kim M.J., Yoo S.J., Jang J.H., Kim H.-J. (2015). Highly efficient and durable TiN nanofiber electrocatalyst supports. Nanoscale.

[27] Wang C., Chi M., Li D., Strmcnik D., Van Der Vliet D., Wang G., Komanicky V., Chang K.-C., Paulikas A.P., Tripkovic D. (2011). Design and Synthesis of Bimetallic Electrocatalyst with Multilayered Pt-Skin Surfaces. J. Am. Chem. Soc..

[28] Cheng T., Xiao H., Goddard W.A. (2016). Reaction Mechanisms for the Electrochemical Reduction of CO_2_ to CO and Formate on the Cu(100) Surface at 298 K from Quantum Mechanics Free Energy Calculations with Explicit Water. J. Am. Chem. Soc..

[29] Strasser P., Koh S., Anniyev T., Greeley J., More K., Yu C., Liu Z., Kaya S., Nordlund D., Ogasawara H. (2010). Lattice-strain control of the activity in dealloyed core–shell fuel cell catalysts. Nat. Chem..

[30] Delley B. (1990). An all-electron numerical method for solving the local density functional for polyatomic molecules. J. Chem. Phys..

[31] Delley B. (2000). From molecules to solids with the DMol^3^ approach. J. Chem. Phys..

[32] Perdew J.P., Burke K., Ernzerhof M. (1996). Generalized Gradient Approximation Made Simple. Phys. Lett..

[33] Delley B. (2002). Hardness conserving semilocal pseudopotentials. Phys. Rev. B.

[34] Xiao B.B., Liu H.Y., Jiang X.B., Yu Z.D., Jiang Q. (2017). A bifunctional two dimensional TM_3_(HHTP)_2_ monolayer and its variations for oxygen electrode reactions. RSC Adv..

[35] Xiao B., Zhu H., Liu H., Jiang X., Jiang Q. (2018). The Activity Improvement of the TM_3_(hexaiminotriphenylene)_2_ Monolayer for Oxygen Reduction Electrocatalysis: A Density Functional Theory Study. Front. Chem..

[36] Mao J., Li S., Zhang Y., Chu X., Yang Z. (2016). Density functional study on the mechanism for the highly active palladium monolayer supported on titanium carbide for the oxygen reduction reaction. J. Chem. Phys..

[37] Wang Y., Yang Z. (2018). TiC and TiN supported platinum monolayer as high-performance catalysts for CO oxidation: A DFT study. J. Chem. Phys..

[38] Duan Z., Wang G. (2013). Comparison of Reaction Energetics for Oxygen Reduction Reactions on Pt(100), Pt(111), Pt/Ni(100), and Pt/Ni(111) Surfaces: A First-Principles Study. J. Phys. Chem. C.

[39] Wang S., Chu X., Zhang X., Zhang Y., Mao J., Yang Z. (2017). A First-Principles Study of O_2_ Dissociation on Platinum Modified Titanium Carbide: A Possible Efficient Catalyst for the Oxygen Reduction Reaction. J. Phys. Chem. C.

[40] Sha Y., Yu T.H., Merinov B.V., Shirvanian P., Goddard W.A. (2012). Mechanism for Oxygen Reduction Reaction on Pt_3_Ni Alloy Fuel Cell Cathode. J. Phys. Chem. C.

[41] Baran J.D., Grönbeck H., Hellman A. (2014). Analysis of Porphyrines as Catalysts for Electrochemical Reduction of O_2_ and Oxidation of H_2_O. J. Am. Chem. Soc..

[42] Hammer B., Nørskov J. (2000). Theoretical surface science and catalysis—Calculations and concepts. Adv. Catal..

[43] Xin H., Linic S. (2010). Communications: Exceptions to the d-band model of chemisorption on metal surfaces: The dominant role of repulsion between adsorbate states and metal d-states. J. Chem. Phys..

[44] Liu W., Lian J.S., Jiang Q. (2007). Theoretical Study of C_2_H_2_ Adsorbed on Low-Index Cu Surfaces. J. Phys. Chem. C.

[45] Gao G., Bottle S.E., Du A. (2018). Understanding the activity and selectivity of single atom catalysts for hydrogen and oxygen evolution via ab initial study. Catal. Sci. Technol..

[46] Viswanathan V., Heine A.H., Rossmeisl J., Nørskov J.K. (2012). Universality in Oxygen Reduction Electrocatalysis on Metal Surfaces. ACS Catal..

[47] Shao M., Shoemaker K., Peles A., Kaneko K., Protsailo L. (2010). Pt Monolayer on Porous Pd−Cu Alloys as Oxygen Reduction Electrocatalysts†. J. Am. Chem. Soc..

[48] Sha Y., Yu T.H., Merinov B.V., Shirvanian P., Goddard W.A. (2011). Oxygen Hydration Mechanism for the Oxygen Reduction Reaction at Pt and Pd Fuel Cell Catalysts. J. Phys. Chem. Lett..

[49] Yang B., Burch R., Hardacre C., Headdock G., Hu P. (2013). Understanding the Optimal Adsorption Energies for Catalyst Screening in Heterogeneous Catalysis. ACS Catal..

[50] Shang C., Liu Z.-P. (2011). Origin and Activity of Gold Nanoparticles as Aerobic Oxidation Catalysts in Aqueous Solution. J. Am. Chem. Soc..

[51] Greeley J., Stephens I.E.L., Bondarenko A.S., Johansson T.P., Hansen H.A., Jaramillo T.F., Rossmeisl J., Chorkendorff I., Nørskov J.K., Jaramillo T. (2009). Alloys of platinum and early transition metals as oxygen reduction electrocatalysts. Nat. Chem..

[52] Stephens I.E.L., Bondarenko A.S., Grønbjerg U., Rossmeisl J., Chorkendorff I. (2012). Understanding the electrocatalysis of oxygen reduction on platinum and its alloys. Energy Environ. Sci..

[53] Lee D.H., Lee W.J., Lee W.J., Kim S.O., Kim Y.-H. (2011). Theory, Synthesis, and Oxygen Reduction Catalysis of Fe-Porphyrin-Like Carbon Nanotube. Phys. Lett..

[54] Favaro M., Ferrighi L., Fazio G., Colazzo L., Di Valentin C., Durante C., Sedona F., Gennaro A., Agnoli S., Granozzi G. (2015). Single and Multiple Doping in Graphene Quantum Dots: Unraveling the Origin of Selectivity in the Oxygen Reduction Reaction. ACS Catal..

[55] Jia Y., Zhang L., Du A., Gao G., Chen J., Yan X., Brown C.L., Yao X. (2016). Defect Graphene as a Trifunctional Catalyst for Electrochemical Reactions. Adv. Mater..

[56] Man I.C., Su H.-Y., Hansen H.A., Martinez J.I., Inoglu N.G., Kitchin J., Jaramillo T.F., Nørskov J.K., Rossmeisl J., Calle-Vallejo F. (2011). Universality in Oxygen Evolution Electrocatalysis on Oxide Surfaces. ChemCatChem.

[57] Zhang P., Hou X., Liu L., Mi J.-L., Dong M. (2015). Two Dimensional π-Conjugated Metal Bis(dithiolene) Complex Nanosheets as Selective Catalysts for Oxygen Reduction Reaction. J. Phys. Chem. C.

[58] Lang X.Y., Han G.F., Xiao B.B., Gu L., Yang Z.Z., Wen Z., Zhu Y.F., Zhao M., Li J.C., Jiang Q. (2015). Mesostructured Intermetallic Compounds of Platinum and Non-Transition Metals for Enhanced Electrocatalysis of Oxygen Reduction Reaction. Adv. Funct. Mater..

[59] Hunt S.T., Milina M., Alba-Rubio A.C., Hendon C.H., Dumesic J.A., Román-Leshkov Y. (2016). Self-assembly of noble metal monolayers on transition metal carbide nanoparticle catalysts. Science.

[60] Tsai H.-C., Lee Y.-J., Merinov B.V., Wu P.-W., Yu T.H., Goddard W.A., Hsieh Y.-C., Wu Y.-H., Chen S.-Y., Adzic R.R. (2015). DFT Study of Oxygen Reduction Reaction on Os/Pt Core–Shell Catalysts Validated by Electrochemical Experiment. ACS Catal..

[61] Wang J.X., Inada H., Wu L., Zhu Y., Choi Y.M., Liu P., Zhou W.-P., Adzic R.R. (2009). Oxygen Reduction on Well-Defined Core−Shell Nanocatalysts: Particle Size, Facet, and Pt Shell Thickness Effects. J. Am. Chem. Soc..

